# Inhibition of Acute Graft-versus-Host Disease with Retention of Graft-versus-Tumor Effects by Dimethyl Fumarate

**DOI:** 10.3389/fimmu.2017.01605

**Published:** 2017-11-20

**Authors:** Jingjing Han, Shoubao Ma, Huanle Gong, Shuangzhu Liu, Lei Lei, Bo Hu, Yang Xu, Haiyan Liu, Depei Wu

**Affiliations:** ^1^Institute of Blood and Marrow Transplantation, Soochow University, Suzhou, China; ^2^Jiangsu Institute of Hematology, The First Affiliated Hospital of Soochow University, Suzhou, China; ^3^Collaborative Innovation Center of Hematology, Soochow University, Suzhou, China; ^4^Immunology Programme, Department of Microbiology and Immunology, Life Sciences Institute, National University of Singapore, Singapore, Singapore

**Keywords:** acute graft-versus-host disease, graft-versus-leukemia, Nrf2, dimethyl fumarate, Treg cells

## Abstract

Acute graft-versus-host disease (aGVHD) remains a clinical challenge and a major source of morbidity and mortality following allogeneic hematopoietic stem cell transplantation (allo-HSCT). Dimethyl fumarate (DMF), an activator of Nrf2, has been shown to have anti-inflammatory and immunomodulatory properties without significant immunosuppression. We therefore hypothesized that DMF could be potentially harnessed for the treatment of aGVHD with retention of graft-versus-tumor effect. In this study, we showed that DMF significantly inhibited alloreactive T cell responses *in vitro* in mixed lymphocyte reaction assay. Administration of DMF significantly alleviated the severity, histological damage, and the overall mortality of aGVHD in an MHC-mismatched aGVHD model. DMF administration reduced the activation and effector function of donor T cells *in vitro* and *in vivo*. In addition, DMF treatment upregulated antioxidant enzymes heme oxygenase-1 and glutathione S-transferase-α1 expressions. Furthermore, DMF treatment markedly increased the frequencies of Treg cells. Depletion of CD25^+^ cells in DMF recipients aggravated aGVHD mortality compared with IgG control recipients. DMF could promote Treg cell differentiation in a dose dependent manner by upregulating TGF-β expression *in vitro*. Most importantly, DMF administration preserved graft-versus-leukemia effect after bone marrow transplantation. In conclusion, our findings demonstrated DMF as a promising agent for the prevention of aGVHD after allo-HSCT.

## Introduction

Allogeneic hematopoietic stem cell transplantation (allo-HSCT) has become a potential curative treatment for malignant hematological diseases (1). However, the success of an allo-HSCT is frequently limited by life-threatening complications, such as acute graft-versus-host disease (aGVHD) (1). aGVHD is a T cell-mediated disease which is caused by alloreactive donor T cells recognizing and attacking recipient target organs, such as the liver, lungs, intestines and skin (2). Various effector T subsets, Th1, Th2, and Th17, are involved in the pathogenesis of aGVHD (3). They particularly contribute to the initiation and development and of aGVHD, and have been considered as potential targets for the treatment and prevention of aGVHD (3). Currently, therapy of established aGVHD is still dependent on corticosteroids, despite their limited efficacy and considerable toxicity (2). Therefore, development of novel therapies will be critical for the prevention and treatment of aGVHD.

It is previously noticed that conditioning regimens including high-dose chemotherapy and radiation therapy generally result in the formation of reactive oxygen species (ROS) in allo-HSCT patients, which triggers inflammatory response and tissue injury, and plays an important role in the development of aGVHD (4–6). Therefore, appropriate control of oxidative stress, particularly ROS production, is crucial for effectively managing aGVHD. The transcription factor nuclear factor erythroid 2-related factor 2 (Nrf2) is the “master regulator” of the antioxidant response. Upon exposure to ROS, Nrf2 translocates to the nucleus, binds to antioxidant response elements (AREs) in combination with hundreds of genes located in the promoter region to confer antioxidant protective effects (7). Therefore, it is proposed that Nrf2 activation can scavenge oxygen free radicals produced by conditioning regimens of allo-HSCT, then therefore, inhibit oxidative stress damage to organs and tissues.

Dimethyl fumarate (DMF) was first proposed by a German chemist, Walter Schweckendiek, in 1959, initially for the treatment of psoriasis (8). It was then developed as an oral capsule for the treatment of adults with relapsing forms of multiple sclerosis (MS) (trade name Tecfidera) on March 27, 2013 (9). The pharmacological properties of DMF include the activation of Nrf2-dependent antioxidant response and inhibition of NF-κB pathway (10). On the one hand, DMF activates Nrf2 and induces the expression of many antioxidant defense enzymes, such as the sentinel cytoprotectant heme oxygenase-1 (HO-1), NAD(P)H quinone oxidoreductase-1, and glutathione S-transferase (GST) (11, 12). On the other hand, Nrf2 activation can simultaneously inhibit the NF-κB signaling pathway, and consequently modulate inflammatory cytokines and chemokine production, such as IL-1, IL-2, IL-6, iNOS, IFN-γ, as well as CCL2 and CXCL10 (11, 13, 14). Moreover, DMF could inhibit Th1 polarization and promote Th2 differentiation (15, 16), inhibit the maturation and function of dendritic cells (DCs), as well as the subsequent DC-mediated Th1 and Th17 cell responses (17). Furthermore, DMF also suppresses CCL2-induced chemotaxis of human monocytes (18), inhibits lipopolysaccharide (LPS) induced proinflammatory cytokine production in macrophages (19). Therefore, through a combination of Nrf2 activation and NF-κB signaling inhibition, DMF has been shown to have anti-inflammatory, anti-oxidative, and immunomodulatory properties without significant immunosuppression.

Based on the immunomodulatory effect of DMF, we hypothesize that DMF could have the potential for the treatment of aGVHD with retention of graft-versus-tumor effect after allo-HSCT. In this study, by using murine models of aGVHD and GVL, we showed that Nrf2 activation by DMF treatment significantly reduced aGVHD without impairing GVL effect. The protective role of DMF in aGVHD was associated with increased donor Tregs and reduced T cells infiltration and activation in aGVHD target organs. Our findings suggest that DMF can be used for the prevention and treatment of aGVHD.

## Materials and Methods

### Mice and Leukemia Cell Line

Female C57BL/6 (H-2^b^) and BALB/C (H-2^d^) mice were purchased from Shanghai Laboratory Animal Center (Shanghai, China). All mice were maintained in a specific pathogen-free room at Animal Facilities of Soochow University. Experiments were carried out and approved according to the guidelines of the animal care and use committee at Soochow University. A20 lymphoma cells were purchased from American Type Culture Collection (Rockville, MD, USA). Cells were cultured at 37°C in a 5% CO_2_ incubator in RPMI 1640 culture media supplemented with 10% fetal bovine serum (Biological Industries, Co., Haemek, Israel). For bioluminescent imaging, stable luciferase-expressing A20 cells (A20-luc) were generated in our laboratory.

### aGVHD and GVL Models

Murine aGVHD and GVL models were induced as described previously (20, 21). Briefly, BALB/c mice were given lethally 650 cGy (one dose) total body irradiation from X-ray, irradiated BALB/c mice was transplanted with 1 × 10^7^ C57BL/6 bone marrow cells and 5 × 10^6^ C57BL/6 spleen cells *via* the tail vein. DMF (30 mg/kg body weight, Item No. 50744, Sigma-Aldrich, USA) was administrated to the recipient mice by gavage once daily starting from day −3 to day 3 after bone marrow transplantation (BMT). 0.8% methocel (Sigma-Aldrich Fluka, USA) at the same volume was used as vehicle control. For GVL model, 1 × 10^6^ A20-luc cells were added to bone marrow graft as mentioned above, and injected into lethally irradiated BALB/c mice. *In vivo* bioluminescence imaging was performed as described previously (20). Briefly, mice were given an intraperitoneal injection of 200 µg firefly luciferin and then anesthetized and imaged using Xenogen, IVIS 100 Bioluminescent Imaging System (Caliper Life Sciences, Hopkinton, MA, USA). Treg depletion was performed as described previously (22, 23). Briefly, lethally irradiated BALB/c mice were transplanted with 5 × 10^6^ TCD-BM plus 1 × 10^6^ total spleen T cells or CD25-depleted T cells from B6 mice, DMF was administered to these recipients, with vehicle treatment as control. T cell depletion was performed by anti-Thy1.2 mAb (30H12, Biolegend, USA) and rabbit complement (24). T cell purification was performed by using mouse T cell isolation kit (catalog #19851, Stemcell technologies, Vancouver, BC, USA), CD25 depletion was performed by using mouse CD25 regulatory T cell positive selection kit (catalog #18782, Stemcell technologies, USA) according to the manufacturer’s protocols. Unlabeled CD25 negative cells were collected. CD25 depletion efficiency was confirmed by FACS. The recipients were monitored daily for survival and every three days for body weight changes and clinical signs of GVHD. The severity of GVHD was assessed using a GVHD scoring system as described previously (20, 21).

### Histopathologic Analysis

Fourteen days after transplantation, liver, lung, small intestine and skin were obtained from the transplanted recipients and fixed in 10% formalin. Samples were then embedded in paraffin, sectioned were stained with hematoxylin and eosin. Tissue damage was assessed based on a semiquantitative scoring system as described previously (25, 26).

### Mixed Lymphocyte Reaction (MLR) and Cytotoxicity Assay

Mixed lymphocyte reaction assay was performed as described previously (27). Briefly, responder T cells were isolated from spleen of C57BL/6 mice by mouse T cell enrichment kit (StemCell Technologies, Vancouver, BC, Canada). Stimulators were DCs from BALB/c cells. BM-derived DCs were generated and expanded from BALB/c mice with GM-CSF (10 ng/ml) and IL-4 (10 ng/ml) for 7 days. DCs were pretreated with DMF or DMSO for 24 h, then washed twice with PBS. 1 × 10^4^ DCs treated as above were irradiated (30 Gy) and cocultured with 1 × 10^5^ allogeneic T cells in U-bottom microwell plates. 5 days later, tritiated thymidine (^3^H-TdR, 1 mCi/well) (Shanghai Institute of Physics, Chinese Academy of Sciences) were added to the culture for 16–18 h prior to harvesting and were counted on a β-plate reader (PerkinElmer Instruments, Meriden, CT, USA). Cytokines in the supernatants were collected and measured by ELISA. In some experiments, T cells were labeled with CellTrace CFSE (5 µmol/L, Invitrogen) according to the manufacturer’s protocol. The CFSE dilution was examined by flow cytometry. For *ex vivo* MLR, splenocytes from transplanted recipients 14 days after BMT were as responders, irradiated splenocytes from BALB/c mice were as stimulators. Cytotoxicity assays were performed as described previously (20). Splenocytes from transplanted recipients 14 days after BMT were used as killing cells, and their killing ability of A20 targets was measured using CytoTox 96 nonradioactive cytotoxicity assay kit (Promega, Fitchburg, WI, USA).

### Cell Preparation and Flow Cytometry

The procedure for isolating single-cell suspensions from spleens has been described previously (21). Antibodies against CD3, CD4, CD8, CD11c, CD25, CD40, CD44, CD69, CD86, PD-1, PD-L1, IFN-γ, H-2k^b^, H-2k^d^ used in this study were all purchased from BioLegend (San Diego, CA, USA). For cell surface staining, cell samples were stained with fluorescent dye-conjugated mAb for 20 min at 4°C in the presence of FcR-Block. For intracellular cytokine staining, cells were stimulated for 5 h with PMA (50 ng/ml) and ionomycin (500 ng/ml) in the presence of brefeldin A (10 µg/ml). Cells were harvested, washed, and stained with surface molecule antibodies in the presence of FcR-Block (eBioscience, San Diego, CA, USA). After the wash, cells were then fixed using CytoFix/CytoPerm buffer (BD Biosciences, USA) and stained with antibodies against intracellular cytokines or isotype control on ice for 30 min. Intracellular staining for FoxP3 was performed by using a Foxp3 staining kit (eBioscience, San Diego, CA, USA). Data were acquired on a NovoCyte Flow cytometer (ACEA Biosciences, San Diego, CA, USA) and analyzed using Flowjo software (FlowJo, Ashland, OR, USA).

### ELISA

Blood samples were obtained from recipients 14 days after BMT, serum was separated by centrifugation and was stored at −80°C. Culture supernatants were collected at indicated time by centrifugation. The levels of IL-2, IL-6, IFN-γ, TNF-α, TGF-β were examined by ELISA kit according to the manufacturer’s instructions (R&D system, Minneapolis, MN, USA).

#### Immunofluorescent Microscopy

For examining Nrf2 nuclear translocation, CD3^+^T cells were isolated from spleen of C57BL/6 mice and activated by plate bound anti-CD3 (5 µg/ml) and anti-CD28 (1 µg/ml) in the presence of DMF or DMSO for 3 h. The cells were harvested and fixed in 4% paraformaldehyde for 15 min, then permeabilized with 0.2% Triton X100 for 10 min, and blocked with 2% BSA for 30 min. Sample were incubated overnight with an anti-Nrf2 antibody (sc-13032, Santa Cruz, CA, USA) in 0.5% BSA. After three washes with PBS, cells were stained with Alexa Fluor 488 goat antirabbit IgG (Molecular Probes, USA). Cell nuclei were stained with DAPI. The fluorescent images were captured with the Leica DMi8 confocal microscope (Leica, Wetzlar, Germany).

### Real-time PCR

Total RNA were extracted with TRIzol reagent (Takara, Japan) from aGVHD targets organs according to the manufacturer’s instructions. Transcription levels of Nrf2, Keap1, HO-1, GST-α1, IL-1β, IL-2, IL-6, IFN-γ genes were analyzed by real-time PCR using SYBR Green Master Mix (Applied Biosystems, Warrington, UK). The primers used were: Nrf2, Forward 5′-TAGATGACCATGAGTCGCTTGC-3′, reverse 5′-GCCAAACTTGCTCCATGTCC-3′; keap1, forward 5′-TGCCCCTGTGGTCAAAGTG-3′, reverse 5′-GGTTCGGTTACCGTCCTGC-3′; HO-1, forward 5′-AAGCCGAGAATGCTGAGTTCA-3′, reverse 5′-GCCGTGTAGATATGGTACAAGGA-3′; GST-α1, forward 5′-AAGCCCGTGCTTCACTACTTC-3′, reverse 5′-GGGCACTTGGTCAAACATCAAA-3′; β-actin, forward 5′-ATCTGGCACCACACCTTC-3′, reverse 5′-AGCCAGGTCCAGACGCA-3′. The relative expression of the gene was quantified using the comparative 2^−ΔΔCt^ method relative to the housekeeping gene β-actin.

### ROS Detection

CD3^+^ T cells were isolated from spleen of C57BL/6 mice and activated by plate bound anti-CD3 (5 µg/ml) and anti-CD28 (1 µg/ml) in the presence of DMF or DMSO for 12 h. ROS levels were examined by Reactive Oxygen Species Assay Kit according to the manufacturer’s instructions (Beyotime, Shanghai, China). Briefly, cells were harvested and washed three times using RPMI-1640 media, 2′-7′-dichlorofluoresci1n diacetate was added at a final concentration of 10 µM to the cells. After a 20-min incubation at 37°C, cells were washed and resuspended in PBS. Data were acquired on a NovoCyte Flow cytometer and analyzed using Flowjo software.

### Cell Proliferation and Apoptosis

For cell proliferation, 2 × 10^4^ A20 cells were seeded into 96-well plates and treated with DMF or DMSO for 48 h. Then 10 µl of CCK-8 solution was added to each well and incubated for additional 2 h, the absorbance was measured at 450 nm. Cell apoptosis was evaluated using a Annexin V Apoptosis Detection Kit (eBioscience, San Diego, CA, USA) according to the manufacturer’s instructions. Briefly, 48 h after DMF treatment, the cells were washed with PBS and resuspended in 500 µl binding buffer with 5 µl annexin V and 10 µl propidium iodide. Data were acquired by flow cytometry and analyzed using Flowjo software.

### Statistical Analysis

Survival data were analyzed by log-rank test and Kaplan-Meier survival curves were generated using GraphPad Prism version 5 (GraphPad 6.0, San Diego, CA, USA). The data were expressed as the mean ± SD. Two-tailed Student’s *t*-test was used for statistical comparison between two groups. One-way ANOVA with Dunnet’s test was used for multiple comparisons. The significance levels are marked *P < 0.05; **P < 0.01; ***P < 0.001.

## Results

### DMF Inhibits Alloreactive T Cell Responses *In Vitro*

**Figure 1:**
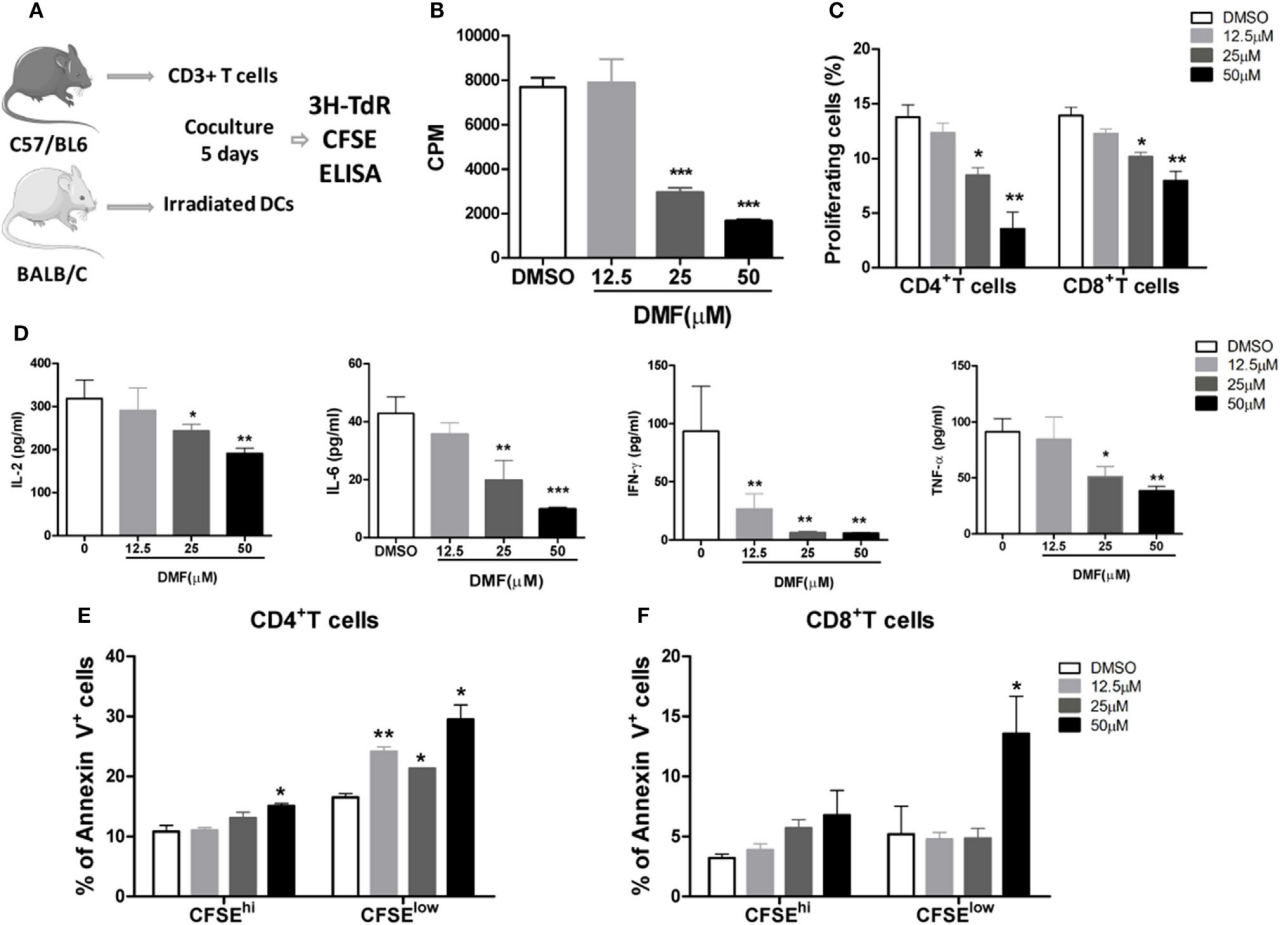
Dimethyl fumarate (DMF) inhibits alloreactive T cell responses *in vitro*. BMDCs from BALB/c mice were cocultured with allogeneic splenic CD3^+^ T cells purified from C57BL/6 mice, in the presence of the DMF **(A)**, 5 days later, cell proliferation was evaluated by ^3^H-TdR **(B)** or CFSE **(C)**. The levels of proinflammatory cytokines IL-2, IL-6, IFN-γ, and TNF-α in the supernatants were examined by ELISA **(D)**. Cell apoptosis of alloreactive CD4^+^ T cells and CD8^+^ T cells was examined by Annexin V staining **(E,F)**. Data shown are mean ± SD. *P < 0.05; **P < 0.01; ***P < 0.001, compared with DMSO group (ANOVA with Dunnett’s test).

Dimethyl fumarate has been demonstrated to potently inhibit NF-κB activity while promote Nrf2 activation. Therefore, we investigated the impact of DMF on alloreactive T cell responses in MLR assays. BMDCs from BALB/c mice were cultured with allogeneic splenic CD3^+^T cells purified from C57BL/6 mice, in the presence of the Nrf2 activator DMF (Figure 1A). The results showed that DMF significantly inhibited the proliferation of alloreactive T cells in a dose-dependent manner on day 5 determined by ^3^H-TdR and CFSE dye dilution (Figures 1B,C). Consistent with the T cell proliferation results, cytokine analysis showed that proinflammatory cytokines IL-2, IL-6, IFN-γ, and TNF-α production were significantly decreased in a dose-dependent manner upon DMF treatment (Figure 1D). In addition, cell apoptosis assay showed that the apoptosis of alloreactive CD4^+^ T cells were significantly increased in the presence of DMF (Figures 1E,F). More interesting, the apoptotic cells were mainly CFSE^low^ populations. To address whether DMF has a direct effect on T cells, we performed a T cell activation assay with anti-CD3/CD28 stimulation, the result showed that DMF could significantly inhibit T cells proliferation directly (Figure S1A in Supplementary Material). Additionally, we examined the effect of DMF on antigen presenting cells (APC). We cultured bone marrow DCs and then treated with DMF. The data showed that DMF had no effect on DC maturation, however, when DCs were preactivated by LPS, DMF could reduce CD80, CD86, and CD40 expression in a dose-dependent manner (Figure S1B–D in Supplementary Material). Similar results were observed in IL-6 and TNF-α expression (Figure S1E,F in Supplementary Material). Taken together, the results demonstrated that DMF could inhibit alloreactive T responses by suppressing cell proliferation and inducing cell apoptosis *in vitro*, through both direct effects on T cells and indirect effects on DCs.

### DMF Administration Alleviates aGVHD in Mice

**Figure 2:**
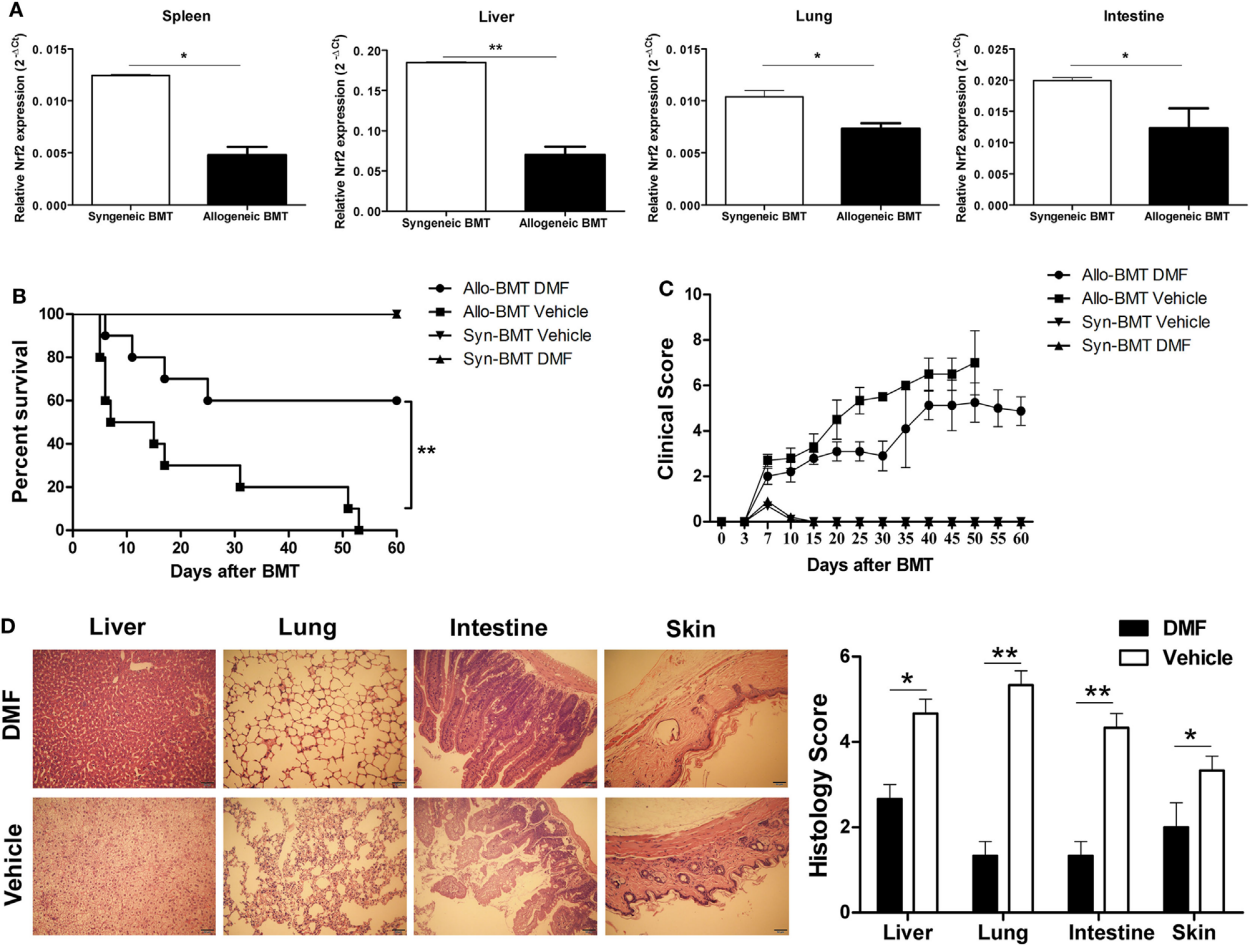
Dimethyl fumarate (DMF) administration alleviates acute graft-versus-host disease (aGVHD) in mice. Irradiated BALB/c mice was transplanted with 1 × 10^7^ C57BL/6 bone marrow cells and 5 × 10^6^ C57BL/6 spleen cells (allogeneic) or 1 × 10^7^ BALB/c bone marrow cells and 5 × 10^6^ BALB/c spleen cells (syngeneic). The mRNA level of Nrf2 in spleen, liver, lung and intestine of allogeneic or syngeneic bone marrow transplantation (BMT) mice was examined by qRT-PCR **(A)**. DMF (30 mg/kg body weight) was administrated to the allogeneic recipient mice by gavage once daily starting from day −3 to day 3 after BMT. DMF treatment significantly prolongs survival (log rank, *P* = 0.005) **(B)** and reduced aGVHD severity **(C)** compared with vehicle control. Histological analysis revealed that there was decreased pathological damage in the liver, lung, intestine and skin of recipients receiving DMF 14 days after BMT **(D)**. Survival data were analyzed by log-rank test and Kaplan-Meier survival curves were generated using GraphPad Prism. Data shown are mean ± SD. *P < 0.05; **P < 0.01; ***P < 0.001, compared with vehicle group (Student’s *t*-test).

To determine the possible role of Nrf2 activation in aGVHD, we first examined the Nrf2 expression after allo-HSCT. We observed that Nrf2 mRNA levels were significantly decreased in the spleen, liver, lung and intestine of allogeneic BMT mice compared to those of syngeneic control animals (Figure 2A), suggesting that Nrf2 suppression could be involved in the pathogenesis of aGVHD. We then evaluated the potential impact of DMF on the aGVHD development *in vivo* by using a murine aGVHD model. As shown in Figure 2B, DMF treatment significantly prolonged survival (log rank, *P* = 0.005) of the hosts following allo-HSCT (Figure 2B). In addition, aGVHD scores were reduced in DMF-treated mice compared with vehicle control recipients (Figure 2C). Histological analysis revealed that there was decreased pathological damage in the liver, lung, intestine and skin of recipients receiving DMF 14 days after allo-HSCT (Figure 2D). Similar protective effect was observed when the dose of DMF was changed to 60 mg/kg from day −3 to day +3 post-allo-HSCT (data not shown). Moreover, DMF administration did not affect donor chimerism 14 days posttransplant (data not shown). Therefore, these data suggested that Nrf2 activation by DMF treatment *in vivo* significantly improved aGVHD outcomes as evidenced by prolonged survival and reduced aGVHD scores following allo-HSCT.

### DMF Administration Reduces Activation and Effector Function of Donor T Cells and Upregulates Antioxidant Response

**Figure 3:**
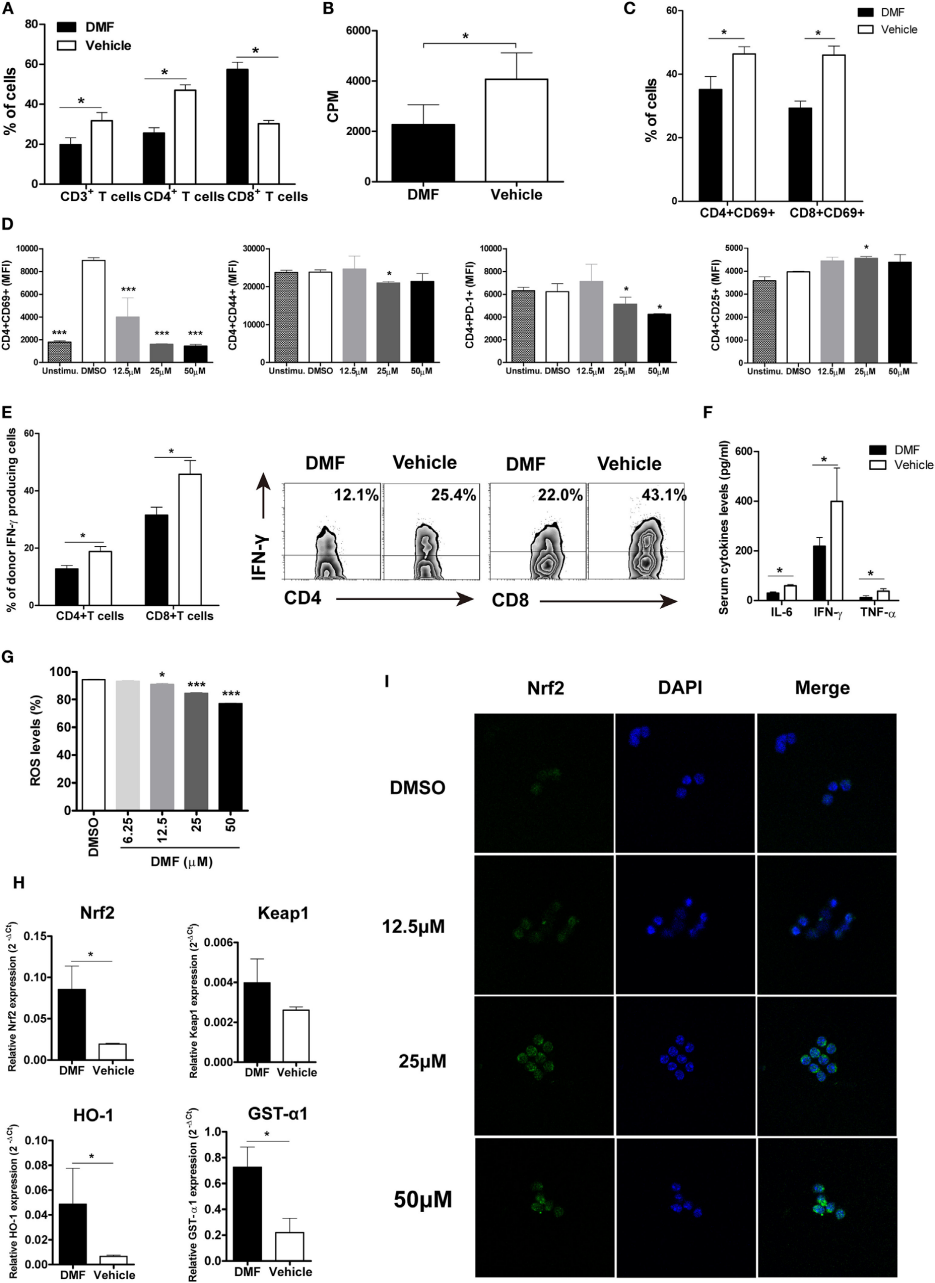
Dimethyl fumarate (DMF) administration reduces activation and effector function of donor T cells and upregulates antioxidant response. 14 days after bone marrow transplantation (BMT), the percentages of donor T cells in spleen were analyzed by FACS **(A)**. *Ex vivo* mixed lymphocyte reaction (MLR) was performed by coculturing splenocytes from transplanted recipients 14 days after BMT with irradiated splenocytes from BALB/c mice. Cell proliferation was evaluated by ^3^H-TdR **(B)**. CD69 expression on CD4^+^ and CD8^+^T cells of recipients mice 14 days after BMT was examined by FACS **(C)**. Purified CD3^+^T cells were activated by anti-CD3/CD28 *in vitro* and treated with DMF. The activation markers CD69, CD44, CD25, and costimulatory molecule PD-1 levels were analyzed 24 h after treatment **(D)**. IFN-γ production by CD4^+^ and CD8^+^T cells of recipients mice 14 days after BMT was examined by Intracellular staining **(E)**. Serum levels of proinflammatory cytokines IL-6, IFN-γ, and TNF-α were examined by ELISA **(F)**. Purified CD3^+^T were activated by anti-CD3/CD28 *in vitro* and treated with DMF for 12 h, reactive oxygen species (ROS) levels were examined by Reactive Oxygen Species Assay Kit **(G)**. The Nrf2, keap 1, and antioxidant defense enzymes HO-1 and GST-α1 mRNA expressions in the spleen were examined by qRT-PCR 14 days after BMT **(H)**. **(I)** CD3^+^T cells were isolated from spleen of C57BL/6 mice and activated by plate bound anti-CD3 (5 µg/ml) and anti-CD28 (1 µg/ml) in the presence of DMF or DMSO for 3 h. Nrf2 nuclear translocation was exmianed by immunofluorescent assay. Data shown are mean ± SD. *P < 0.05; **P < 0.01; ***P < 0.001, compared with vehicle group (Student’s *t*-test), or DMSO group (ANOVA with Dunnett’s test).

To investigate the potential mechanisms responsible for reduced aGVHD severity by DMF treatment, we analyzed the alloreactive T cell responses *in vivo*. On day 14 after allo-HSCT, decreased infiltration of donor CD3^+^ T and CD4^+^ T cells was observed in spleen of DMF-treated recipients compared with vehicle control recipients (Figure 3A). The *ex vivo* MLR assay further demonstrated that DMF treatment significantly reduced donor T cell alloreactivity (Figure 3B). In addition, both donor CD4^+^ T and CD8^+^ T cells had a reduced activation phenotype indicated by CD69 levels in the hosts treated with DMF (Figure 3C). Furthermore, purified CD4^+^ T cells were activated *in vitro* by anti-CD3/CD28, and DMF treatment significantly inhibited T cell activation and downregulated CD69, CD44, as well as PD-1 expressions. The CD25 levels, however, were slightly upregulated by DMF treatment (Figure 3D). Intracellular staining revealed that the frequencies of IFN-γ-producing donor CD4^+^ T and CD8^+^ T cells in spleen were significantly decreased in recipients given DMF (Figure 3E). Analysis of serum samples on day 14 following allo-HSCT showed that the levels of proinflammatory cytokines IL-6, IFN-γ as well as TNF-α were significantly downregulated in DMF-treated recipients compared with vehicle control recipients (Figure 3F).

Since DMF is a potent activator of Nrf2 and can induce the Nrf2-dependent antioxidant response, we then examined the ROS production and antioxidant genes expression. We found that DMF could significantly inhibit ROS production by activated T cells in a dose-dependent manner *in vitro* (Figure 3G). Moreover, the Nrf2 levels and antioxidant defense enzymes HO-1 and GST-α1 expressions were upregulated in recipients treated with DMF (Figure 3H). We then explored the effect of DMF on nuclear translocation of Nrf2, which has been demonstrated as a major mechanism of function for DMF. We observed a dose-dependent effect of DMF on nuclear translocation of Nrf2 in the CD3^+^ T cells by immunofluorescent staining (Figure 3I). Taken together, these results suggested that DMF could inhibit donor T cell alloreactivity, and production of proinflammatory cytokines, as well as upregulate antioxidant enzymes, which led to the alleviation of aGVHD severity.

### DMF Inhibits aGVHD by Promoting Treg Cells

**Figure 4:**
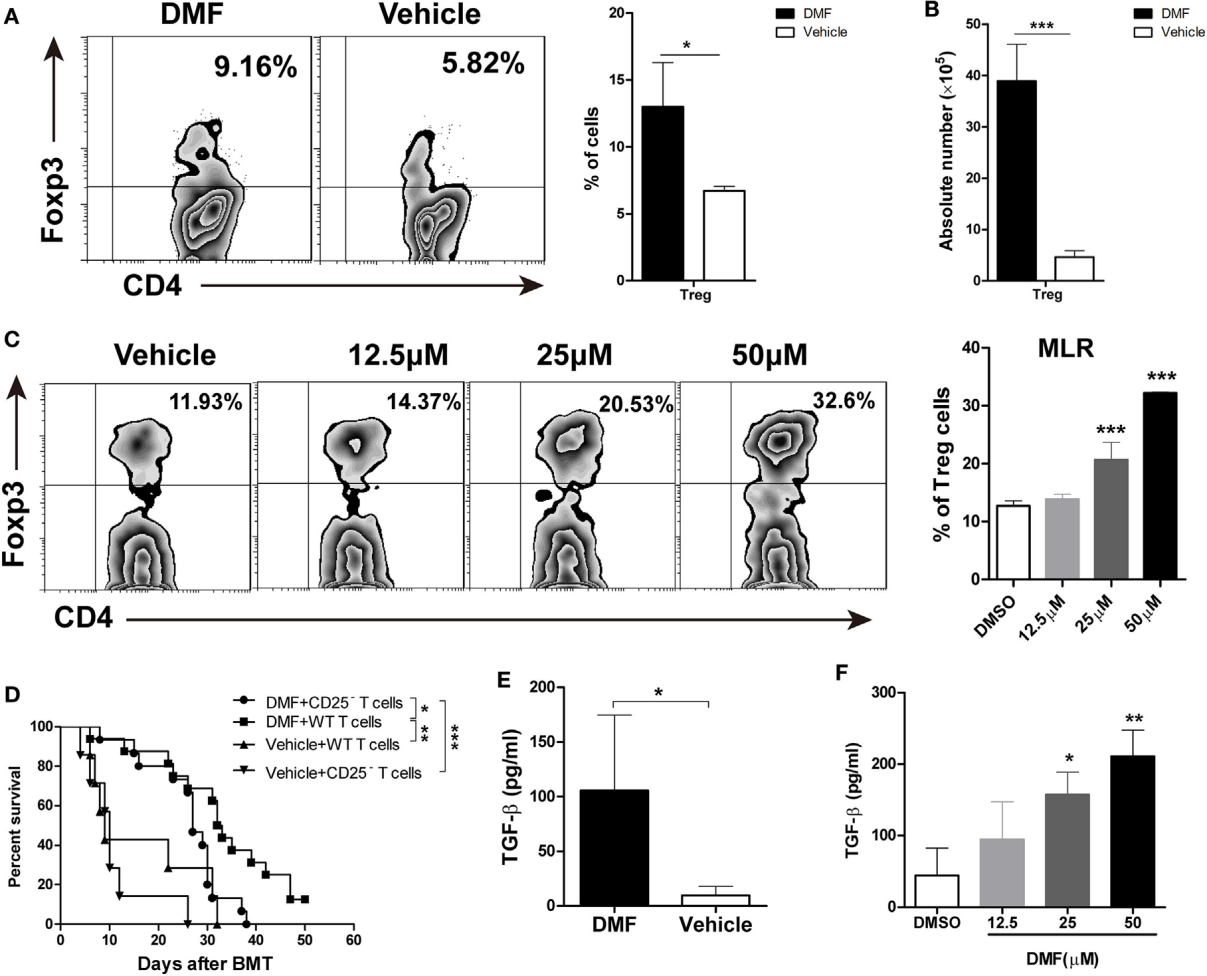
Dimethyl fumarate (DMF) inhibits acute graft-versus-host disease (aGVHD) by promoting Treg cells *in vitro* and *in vivo*. The frequencies and absolute number of CD4^+^Foxp3^+^ Treg cells in spleen on day 14 after bone marrow transplantation (BMT) were examined by FACS **(A,B)**. Purified CD4^+^ T were activated by anti-CD3/CD28 in the presence of TGF-β (2 ng/ml) and IL-2 (50 U/ml) to induce Treg cells development *in vitro*, The effect of DMF on Treg cells differentiation was measured by FACS 4 days after Treg polarization **(C)**. Lethally irradiated BALB/c mice were transplanted with 5 × 10^6^ TCD-BM plus 1 × 10^6^ total spleen T cells or CD25-depleted T cells from B6 mice. Depletion of CD25^+^ cells in DMF recipients aggravated aGVHD mortality compared with IgG control recipients **(D)**. TGF-β levels in serum of aGVHD mice and MLR culture supernatants were measured by qRT-PCR **(E,F)**. Survival data were analyzed by log-rank test and Kaplan-Meier survival curves were generated using GraphPad Prism. Data shown are mean ± SD. *P < 0.05; **P < 0.01; ***P < 0.001, compared with DMSO group or vehicle group (Student’s *t*-test), or DMSO group (ANOVA with Dunnett’s test).

Dimethyl fumarate upregulated CD25 expression on CD4 T cells, suggesting that DMF may promote the generation of Treg cells. We examined the frequencies and absolute numbers of CD4^+^Foxp3^+^ Treg cells in spleen on day 14 after allo-HSCT. As shown in Figures 4A,B, the frequencies, as well as the numbers of Treg cells in spleen were significantly increased in DMF-treated compared with vehicle control recipients, suggesting that DMF could increase Treg cells *in vivo*. *In vitro* MLR assay showed that DMF promoted Treg cell differentiation in a dose-dependent manner (Figure 4C). To further confirm that the effect of DMF on aGVHD was partially dependent on the promotion of Treg cells, mice were injected with anti-CD25 antibody to deplete Treg cells *in vivo*. As shown in Figure 4D, depletion of CD25^+^ cells in DMF recipients aggravated aGVHD mortality compared with IgG control recipients, while it had no effect on vehicle treatment recipients. TGF-β has been known to promote Treg generation, we therefore examined the TGF-β levels in serum of GVHD mice and in MLR culture supernatants. Date shown that TGF-β was increased in DMF recipients (Figure 4E) and upregulated upon DMF treatment *in vitro* (Figure 4F). Therefore, these results suggested that the protective effect of DMF treatment on aGVHD was at least partially dependent on the promotion of Treg cells.

### DMF Administration Preserves GVL Effect after allo-HSCT

**Figure 5:**
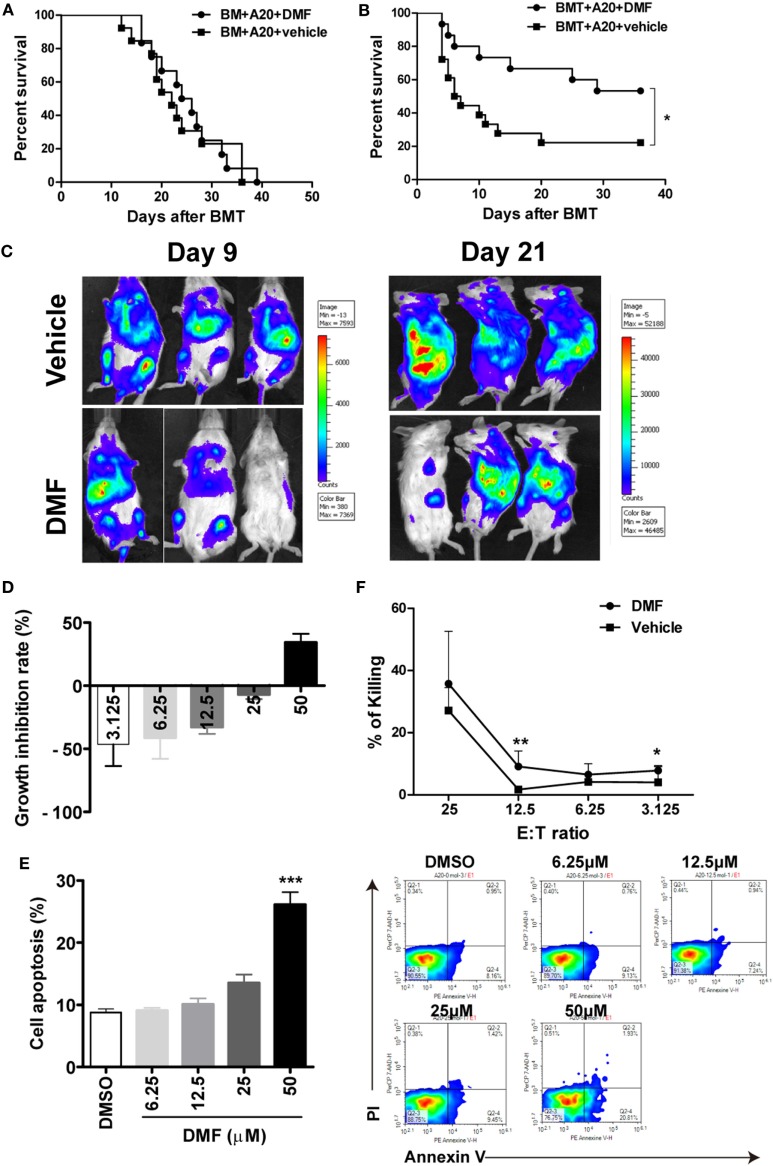
Dimethyl fumarate (DMF) administration preserves GVL effect after bone marrow transplantation (BMT). Mice were transplated with BM or BM and spleen plus A20-luc to establish GVL model. Mice transplanted with allo-BM alone plus A20-luc leukemia cells all died from leukemia regardless of whether DMF was administered **(A)**. DMF administration to mice receiving allo-BMT plus A20-luc showed prolonged survival time and reduced tumor burden compared with mice receiving vehicle control **(B,C)**. The effects of DMF on A20 cell proliferation **(D)** and apoptosis **(E)** were examined by CCK-8 assay and Annexin V/PI staining. Splenocytes from transplanted recipients 14 days after BMT were used as killing cells, and their killing ability of A20 targets was measured using CytoTox 96 nonradioactive cytotoxicity assay kit **(F)**. Survival data were analyzed by log-rank test and Kaplan–Meier survival curves were generated using GraphPad Prism. Data shown are mean ± SD. *P < 0.05; **P < 0.01, compared with DMSO group (ANOVA with Dunnett’s test) or vehicle group (Student’s *t*-test).

To evaluate the impact of DMF administration on GVL effects, aGVHD mice were challenged with A20-luc leukemia cells post-allo-HSCT. As shown in Figure 5A, mice transplanted with allo-BM alone plus A20-luc leukemia cells all died from leukemia within 40 days after transplant, regardless of whether DMF was administered. It has been reported that DMF could inhibit cell proliferation and induce apoptosis in a number of malignant cell lines including myeloid and lymphoid leukemia cell lines. However, the results in Figure 5A suggested that DMF may not directly affect A20 cell growth *in vivo*. DMF administration to mice receiving allo-BM and splenocytes, plus A20-luc showed prolonged survival compared with mice receiving vehicle control (Figure 5B), and low tumor burden was observed in DMF recipients as shown in bioluminescence imaging (Figure 5C), indicating the presence of GVL effect in DMF treated mice. The cell proliferation and apoptosis assay showed that low concentrations of DMF could not affect tumor growth and apoptosis *in vitro*, suggesting that DMF treatment inhibits A20 cell growth only at high concentrations, which may not be reached *in vivo* (Figures 5D,E). We also observed that donor T cells from DMF-treated recipients showed comparable, or even increased CTL killing activity against A20 leukemia cells compared to vehicle controls (Figure 5F). These findings further supported the observations that DMF treatment could preserve GVL effect after allo-HSCT.

## Discussion

Dimethyl fumarate has been shown to have potent anti-inflammatory or immunomodulatory properties without significant immunosuppression. It is effective in treating immune-mediated diseases, including psoriasis, MS as well as colitis (28–30). aGVHD is an immune-mediated disease which resulting from the activation of donor T lymphocytes by host antigen-presenting cell (APC) (2). During aGVHD, donor T cells activated by APCs express multiple immune effector molecules, such as fas ligand, perforin, IL-1β, IFN-γ, and TNF-α, leading to tissue damage (31, 32). We therefore hypothesize that DMF may be a new promising drug for the treatment of aGVHD.

In the present study, we first evaluated the effect of DMF on alloreactive T cell responses in MLR assays. We found that DMF significantly inhibited the proliferation and proinflammatory cytokine production of alloreactive T cells in a dose-dependent manner. In addition, DMF could also induce cell apoptosis of activated alloreactive T cells *in vitro*. In agreement with our results, Joachim et al., showed that DMF could inhibit lymphocyte proliferation and inflammatory cytokine secretion in human peripheral blood mononuclear cells (PBMCs) stimulated with LPS or lectin phytohemagglutinin or in human MLR assays (11). In addition, DMF could block IFN-γ- and LPS-induced Th1 chemokine (CXCL9 and CXCL10) production in a dose-dependent manner in PBMCs (33). DMF has also been shown to decrease adhesion molecule expression, such as CD25, HLA-DR and cutaneous lymphocyte-associated antigens (34). In our study, we also observed that DMF treatment significantly inhibited T cell activation and downregulated CD69, CD44, as well as PD-1 expressions, and IFN-γ production by donor CD4^+^ T and CD8^+^ T cells in spleen.

The maximum tolerated dose of DMF in human patients is 240 mg taken two to three times daily by oral for treatment of psoriasis (35), and MS (36). In preclinical studies, the effective and safe dose of DMF was 30 mg/kg in experimental autoimmune encephalomyelitis (37), experimental colitis (30) and tumor studies (38) *in vivo*. Therefore, based on the previous animal studies, we chose 30 mg/kg DMF in this study. We found, for the first time, that administration of DMF significantly ameliorated aGVHD in an MHC-mismatched BMT model. Interestingly, the administration window of DMF for treatment of aGVHD was −3 to +3 days post-BMT in our study, while the protective effect of DMF on aGVHD was not as effective when DMF was given from day 0 to day 7 (Data not shown), suggesting that DMF could regulate the sensitivity of the GVHD target organ to radiation induced injury. Administration of DMF pre-transplant is necessary to exert its protective effect. However, this notion still need further investigation.

Dimethyl fumarate protects the host from aGVHD may be *via* multiple mechanisms due to its dual role in Nrf2 pathway and NF-κB signaling. DMF treatment of T cells has previously been shown to decrease Th1 cytokine production, including IL-12, IFN-γ, TNF-α, and IL-17, and promote the expression of Th2 cytokines, such as IL-4 and IL-10 (10, 16, 39). In addition, DMF could also inhibit DCs maturation and subsequent DC mediated T cell responses (17). However, DMF metabolite monomethyl fumarate (MMF) can enhance CD56^+^ NK cells function by the upregulation of CD107a and granzyme B (40). In our murine aGVHD model, we found that DMF treatment reduced the proliferation and activation of donor T cells in spleen. Similarly, the IFN-γ expression by donor T cells, as well as the serum level of proinflammatory cytokines, was significantly decreased. Besides immune regulation, DMF also activates the Nrf2-dependent ARE pathway. We found that DMF could significantly inhibit ROS production in a dose-dependent manner in activated T cells. Moreover, the Nrf2 levels and antioxidant defense enzymes HO-1 and GST-α1 expressions were both unregulated in recipients given DMF. The ARE activation is involved in the induction of multiple downstream responses that protect cells from intracellular oxidative stress and injury, as well as modulate c cytokine and chemokine production (41). Therefore, our results suggest that DMF ameliorates aGVHD by modulating donor T cell activation and effector function, as well as upregulating antioxidant enzymes.

In our study, the activation makers on donor T cells were all downregulated upon DMF treatment except CD25. On the contrary, DMF significantly increased CD25 expression on activated T cells. Previous study showed that DMF promoted IL-2 secretion during human MLR (11). However, there is no evidence showing that DMF can regulate Treg cell development. We found that Treg cells were significantly increased in DMF-treated recipients compared with vehicle control in spleen on day 14 after allo-HSCT, accompanied by increased serum levels of TGF-β. *In vitro* MLR assay showed that DMF could promote Treg cell development in a dose-dependent manner. In addition, the depletion of Tregs in DMF recipients aggravated aGVHD mortality compared with IgG control recipients. Thus, promotion of Tregs cell development may be one of the mechanism that DMF inhibits aGVHD. Further studies are needed to explore the molecular mechanism of DMF in regulating Tregs development and function.

Alloreactive T-cells mediating aGVHD are also important for GVL activity, the ultimate goal of allo-HSCT is to separate GVHD from GVL effect. It has been suggested that GVL effect is primarily mediated by donor CD8^+^ T cells and NK cells, whereas CD4^+^ T cells mainly contribute to the development of aGVHD. In our study, we found that DMF administration did not weaken the GVL effect. Meanwhile, we did not observe inhibition of leukemia cell growth *in vitro* and *in vivo* by DMF treatment, suggesting that the antileukemia effect of DMF may be associated with its effect in maintaining cytolytic activity of donor CD8^+^ T cells and NK cells. To support this notion, cytotoxicity assay showed that CTLs from DMF-treated recipients has comparable, or even increased killing activity against leukemia cells compared to vehicle controls. Previous study has shown that DMF metabolite MMF can augment the NK cell lysis of K562 and RAJI leukemia cells through CD107a and Granzyme B (40). In addition, our results found that CD8^+^T cell numbers were not decreased upon DMF treatment, on the contrary, its proportions were significantly increased, which could be the reason for preserved GVL effects. Interestingly, it was reported that DMF treatment resulted in a preferential loss of CD8^+^ T cells compared with CD4^+^ T cells in patients with MS (42–46), while the proportions of Treg cells, circulating CD56(hi) NK cells, monocytes, and DCs were unaffected (43, 47). Although the underlying mechanisms remain largely unknown, recent data suggest that it maybe due to the differential susceptibility of distinct cell subsets to DMF-induced apoptosis (39). However, the differential effects of DMF on various immune cell subsets in different disease models need further investigation.

In conclusion, we provide evidence for the first time that the Nrf2 activator DMF reduces aGVHD with retention of GVL effect. DMF treatment promoted donor Treg development and reduced alloreactive T cells response, as well as upregulated antioxidant enzyme expressions. Our findings demonstrated DMF as a promising agent for the prevention of aGVHD after allo-HSCT.

## Ethics Statement

All animal experiments were carried out and approved according to the guidelines of the animal care and use committee at Soochow University.

## Author Contributions

HL and DW designed the study; SM, JH, and HG performed the experiments; SL, LL, BH, and YX contributed to the experiments; SM analyzed the data; and SM, HL, and DW wrote the manuscript. All authors have discussed and revised the manuscript.

## Conflict of Interest Statement

The authors declare that the research was conducted in the absence of any commercial or financial relationships that could be construed as a potential conflict of interest.

## Supplementary Material

Figure S1 DMF inhibited T cells proliferation and DCs maturation *in vitro*. CD3^+^T cells were isolated from spleen of C57BL/6 mice and activated by plate bound anti-CD3 (5 µg/ml) and anti-CD28 (1 µg/ml) in the presence of DMF or DMSO for 48 h. cell proliferation was measured by ^3^H-TdR **(A)**, Data shown are mean ± SD. ****P* < 0.001, compared with DMSO group (ANOVA with Dunnett’s test). BM-derived DCs were generated and expanded from BALB/c mice with GM-CSF (10 ng/ml) and IL-4 (10 ng/ml). DCs were treated with DMF or DMSO for 24 h in the present or absence of LPS (1 µg/ml), CD80, CD86, and CD40 on the DCs were examined by FACS **(B–D)**. Cytokines IL-6, TNF-α, IFN-γ in the supernatants were collected and measured by ELISA **(E–G)**. Data shown are mean ± SD. ****P* < 0.001 compared with LPS group (ANOVA with Dunnett’s test).
