# Supplementary figures and images for: Inhibition of Acute Graft-versus-Host Disease with Retention of Graft-versus-Tumor Effects by Dimethyl Fumarate
Source: Front Immunol. 2017 Nov 20;8:1605. doi: 10.3389/fimmu.2017.01605 (PMC5702003; doi:10.3389/fimmu.2017.01605)

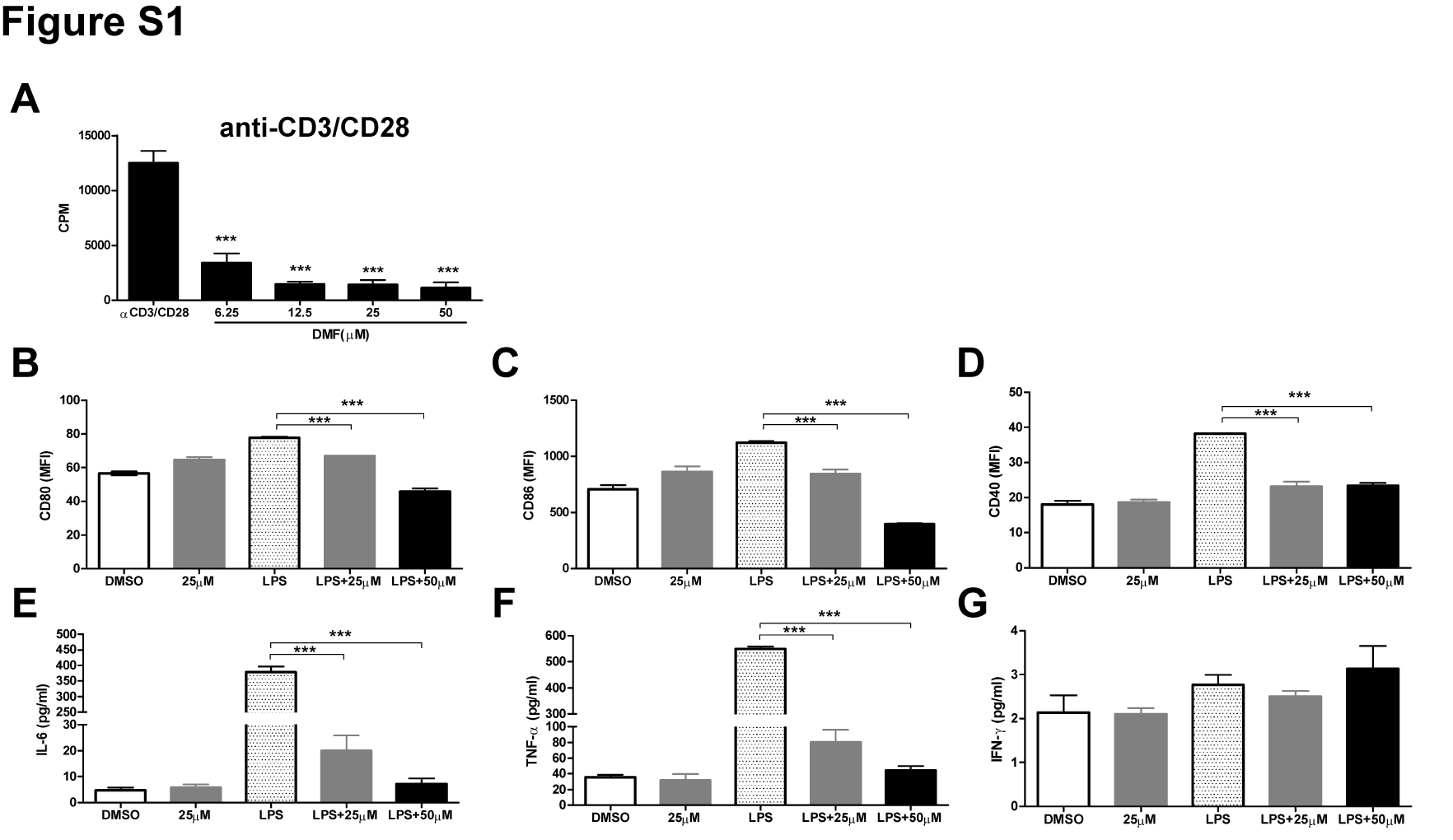

Supplement: Figure S1 — DMF inhibited T cells proliferation and DCs maturation in vitro. CD3+T cells were isolated from spleen of C57BL/6 mice and activated by plate bound anti-CD3 (5 µg/ml) and anti-CD28 (1 µg/ml) in the presence of DMF or DMSO for 48 h. cell proliferation was measured by 3H-TdR (A), Data shown are mean ± SD. ***P < 0.001, compared with DMSO group (ANOVA with Dunnett’s test). BM-derived DCs were generated and expanded from BALB/c mice with GM-CSF (10 ng/ml) and IL-4 (10 ng/ml). DCs were treated with DMF or DMSO for 24 h in the present or absence of LPS (1 µg/ml), CD80, CD86, and CD40 on the DCs were examined by FACS (B–D). Cytokines IL-6, TNF-α, IFN-γ in the supernatants were collected and measured by ELISA (E–G). Data shown are mean ± SD. ***P < 0.001 compared with LPS group (ANOVA with Dunnett’s test). [file image_1.tif]
